# Reimagining writing assessment for the AI era: a systematic review on balancing AI support and authentic skill growth

**DOI:** 10.3389/fpsyg.2026.1809174

**Published:** 2026-05-26

**Authors:** Mohamed Sayed Abdellatif, Mohammed A. Alshehri, Ali Lamouchi, Mohammed Rahmath, Mohamed Ali Nemt-allah

**Affiliations:** 1 Department of Psychology, College of Education in Al-Kharj, Prince Sattam Bin Abdulaziz University, Alkharj, Saudi Arabia; 2 Department of Curriculum and Instruction, College of Education, King Saud University, Riyadh, Saudi Arabia; 3 Department of English Language and Literature, College of Science and Humanities, Prince Sattam Bin Abdulaziz University, Al-Kharj, Saudi Arabia; 4 Department of Computer Engineering and Information, College of Engineering in Wadi Adwassir, Prince Sattam Bin Abdulaziz University, Al-Kharj, Saudi Arabia; 5 Department of Educational Psychology and Statistics, Faculty of Education, Al-Azhar University, Tafhna Al-Ashraf, Egypt

**Keywords:** academic integrity, academic writing assessment, AI literacy, artificial intelligence, generative AI, higher education, stakeholder perceptions

## Abstract

This systematic review synthesizes empirical evidence from 19 studies examining AI (AI) integration in academic writing assessment across higher education contexts. Following PRISMA 2020 guidelines, the review addresses five interconnected research questions regarding AI tool functionality, stakeholder perceptions, student motivations, institutional implementation gaps, and assessment redesign strategies. Findings reveal that generative AI platforms, particularly ChatGPT (88.8% adoption) and Grammarly (67.4%), dominate across writing stages, with students strategically deploying tools based on cognitive complexity levels. Stakeholder perspectives diverge significantly: students value productivity enhancement and accessibility, faculty prioritize academic integrity concerns, and administrators adopt cautious openness with disproportionate resource allocation favoring faculty guidance (67%) over direct student support (17.8%). Student motivations for inappropriate use stem from structural vulnerabilities including time pressures, ethical ambiguity, and institutional policy voids rather than malicious intent. Geographic inequities persist, with Global South institutions facing infrastructure limitations that amplify educational disparities. Promising pedagogical and assessment innovations include process-oriented assessment, oral defenses, multimodal composing, and explicit AI literacy curricula—each targeting the redesign of writing evaluation practices to balance AI assistance with authentic skill development. The review concludes that effective integration requires moving beyond detection-focused approaches toward comprehensive pedagogical redesign grounded in equity, authentic learning, and development of uniquely human capacities for critical judgment and ethical reasoning.

## 1 Introduction

The integration of artificial intelligence (AI) into academic writing has introduced a diverse ecosystem of tools that students and faculty now employ across multiple dimensions of the writing process. Generative chatbots, particularly ChatGPT and other large language models, have become widely adopted for idea generation, drafting, outlining, and providing feedback on academic texts (Barrett and Pack, 2023; Cheng et al., 2025; Dergaa et al., 2023; Escalante et al., 2023; Khlaif et al., 2023; Kim et al., 2024; Nguyen et al., 2024; Song and Song, 2023). Complementing these generative tools, established digital writing assistants such as Grammarly, Quillbot, and Wordtune continue to support grammar checking, style refinement, and paraphrasing (Barrett and Pack, 2023; Gustilo et al., 2024; Escalante et al., 2023). Additionally, machine translation platforms including Google Translate and DeepL facilitate multilingual academic communication (Barrett and Pack, 2023; Ou et al., 2024; Pratiwi et al., 2025), while AI-enhanced reference management and literature mapping tools like Scite AI, Connected Papers, and Litmaps complement traditional platforms such as Zotero and Mendeley (Pratiwi et al., 2025; Weidmann, 2024).

The temporal distribution of AI tool usage reveals distinct patterns across the writing process, shaped in part by the cognitive demands of each stage. Cognitive Load Theory (Sweller, 1988) posits that working memory has a finite capacity, and that learning is optimized when extraneous cognitive demands—those unrelated to the core learning goal—are minimized (Paas et al., 2003). In academic writing, tasks such as spell-checking, grammar correction, punctuation, and surface-level paraphrasing constitute routine mechanical operations in that they do not require domain reasoning or rhetorical judgment; they place demands on working memory without contributing directly to the development of argumentation or disciplinary thinking (Kellogg, 2008).

From a cognitive load theory perspective (Sweller, 1988), this stratified deployment suggests students may be offloading these routine mechanical tasks to AI tools to preserve working memory resources for more complex reasoning—a pattern with both pedagogical promise and risk for authentic skill development (Bauer et al., 2025). This offloading logic provides the conceptual entry point for Bloom’s Taxonomy (Anderson and Krathwohl, 2001), which organizes cognitive work along a hierarchy from lower-order skills—remembering, understanding, and applying—to higher-order skills such as analyzing, evaluating, and creating (Krathwohl, 2002). The mechanical writing tasks described above map onto the lower tiers of this hierarchy, whereas constructing arguments, synthesizing sources, and crafting original claims occupy its upper levels, requiring the kind of sustained deliberate effort that AI delegation may progressively erode (Gerlich, 2025). Bloom’s Taxonomy provides a useful lens for understanding this differentiation: generative AI tools tend to support higher-order cognitive functions such as analysis, evaluation, and creation, while automated writing evaluation tools primarily address lower-order skills including remembering and applying surface-level linguistic conventions (Anderson and Krathwohl, 2001).

Geographic and institutional disparities profoundly shape AI writing tool adoption, with well-resourced institutions in the Global North and major Asian hubs demonstrating significantly faster integration than underfunded universities and Global South regions (Bahroun et al., 2023; Crompton and Burke, 2023; Mustafa et al., 2024; Reina-Parrado et al., 2025). Empirical research concentrates predominantly in North America, Europe, China, and East Asia, reflecting denser institutional capacity and infrastructure (Crompton and Burke, 2023; Kamalov et al., 2023; Mustafa et al., 2024). Within nations, disparities emerge along historical and socioeconomic lines; for instance, historically White South African universities integrate AI more extensively than historically Black institutions facing infrastructural deficits (Maimela and Mbonde, 2025). Global South students report substantially less AI integration in curricula and lower preparedness levels (Busch et al., 2024; Rincón et al., 2025). These inequities stem from limited digital infrastructure, policy gaps, English-centric tool design, and socio-cultural barriers (Chan, 2023; Gustilo et al., 2024; Kalim et al., 2025).

AI integration in writing assessment implicates multiple interdependent stakeholder groups whose perspectives fundamentally shape implementation outcomes. Students represent the primary end-users, directly experiencing AI’s impact on their learning processes, academic integrity concerns, and sense of authorship (Chan and Hu, 2023; Kim et al., 2024; Lin and Chen, 2024; Song and Song, 2023; Wang, 2024), with multilingual learners particularly affected by AI-mediated feedback and language development support (Chen and Gong, 2025; Escalante et al., 2023; Ou et al., 2024). Faculty members occupy a pivotal decision-making role, determining acceptable AI use in assignments while balancing pedagogical benefits against integrity and creativity concerns (Crompton and Burke, 2023; Gustilo et al., 2024; Khlaif et al., 2024). Institutional administrators establish governance frameworks, AI literacy initiatives, and equity policies that structure access and implementation (Chan, 2023; Gustilo et al., 2024; Perkins et al., 2023). Additionally, AI developers and educational technology providers influence assessment practices through system design, affordances, and inherent biases (Escalante et al., 2023; Zhang et al., 2025).

Student decisions regarding appropriate versus inappropriate AI use stem from complex interactions among motivational factors, situational pressures, and ethical frameworks, which this review examines systematically. Social norms, instructor attitudes, and institutional policy clarity further shape these behaviors (Acosta-Enriquez et al., 2024; Chavez et al., 2024; Crawford et al., 2023; Perkins, 2023), while overreliance correlates with diminished critical thinking and creativity (Zhai et al., 2024).

Stakeholder perceptions of AI in writing assessment reveal converging recognition of benefits alongside divergent concerns about integrity and authentic learning. Students widely appreciate AI’s capacity to enhance organization, language quality, feedback, and confidence, particularly among multilingual writers (Chan and Hu, 2023; Escalante et al., 2023; Kim et al., 2024; Ou et al., 2024; Song and Song, 2023; Wang, 2024), yet express concerns about accuracy, overreliance, and loss of authentic voice (Chan, 2024; Johnston et al., 2024; Kim et al., 2024; Ou et al., 2024). Faculty members acknowledge AI’s pedagogical utility while emphasizing threats to assessment validity, distinguishing human from AI-generated text, and maintaining academic integrity (Barrett and Pack, 2023; Dergaa et al., 2023; Gustilo et al., 2024; Ibrahim et al., 2023; Perkins, 2023). Administrators prioritize governance frameworks, policy development, equity considerations, and institutional reputation (Chan, 2023; Lin and Chen, 2024; Perkins, 2023; Yusuf et al., 2024). These divergent priorities underscore the necessity for transparent, collaboratively developed policies that balance innovation with integrity (Chan and Hu, 2023; Gustilo et al., 2024).

AI assistance demonstrates differential effects across cognitive complexity levels, with lower-order skills experiencing more consistent enhancement than higher-order capabilities. Students using AI tools show significant gains in recall, comprehension, and basic application (Abdelmagid et al., 2025; Essien et al., 2024; Zhou et al., 2024), while higher-order skills including critical thinking, analysis, evaluation, and creative synthesis prove more vulnerable to erosion through cognitive offloading (Gerlich, 2025; Zhai et al., 2024). Generative AI performs weakest at Bloom’s create level and struggles with coherent argumentation (Thanh et al., 2023), though intentional pedagogical design can support deeper engagement (Kwan et al., 2025; Zhou et al., 2024). Metacognitive development requires explicit scaffolding rather than emerging naturally from AI interaction (Bauer et al., 2025; Yin et al., 2025; Zhou et al., 2024). Memory retention and autonomous problem-solving face particular risks when students adopt AI as answer-provider rather than thinking partner (Gerlich, 2025; Krsmanović and Deek, 2025; Zhai et al., 2024).

Maintaining assessment validity while leveraging AI support necessitates fundamental redesign rather than incremental adaptation of traditional evaluation approaches. Emerging frameworks emphasize process-oriented assessment that documents iterative revision, reflective decision-making, and AI integration strategies rather than solely evaluating final products (Engeness and Gamlem, 2025; Tseng and Lin, 2024; Wang, 2024). Authentic, cognitively demanding tasks incorporating oral defenses, experiential components, and disciplinary problem-solving help preserve academic integrity while building employability skills (Salinas-Navarro et al., 2024; Sotiriadou et al., 2019; Villarroel et al., 2019). Positioning AI as feedback partner rather than ghostwriter, with structured requirements to critique and adapt AI-generated suggestions, enhances metacognitive engagement and critical evaluation capacities (Akiba and Garte, 2024; Khojasteh et al., 2025; Oates and Johnson, 2025; Song and Song, 2023). Multi-source assessment ecosystems combining self-assessment, peer evaluation, AI analytics, and instructor judgment provide triangulated evidence of competence (Elshall and Badir, 2025; Francis et al., 2025; Tang et al., 2024; Zhang and Zhang, 2022).

Against this complex landscape of opportunities, challenges, and uncertainties surrounding AI integration in academic writing, this systematic review synthesizes empirical evidence from higher education to address five interconnected research questions. It first maps the AI tools used by students and faculty and their functions across writing stages, then examines stakeholder perceptions across linguistic, academic, and institutional contexts. The review also analyzes motivational factors differentiating appropriate from inappropriate AI use, identifies institutional policy and support gaps influencing implementation, and evaluates emerging assessment redesign strategies that uphold validity while leveraging AI for skill development. Through this synthesis, the review advances an evidence based understanding of how writing assessment can be reimagined to balance AI assistance with authentic learning, equity, and academic integrity.

## 2 Materials and methods

### 2.1 Search strategy

This systematic review was conducted in accordance with the Preferred Reporting Items for Systematic Reviews and Meta-Analyses (PRISMA) 2020 guidelines to ensure methodological rigor and transparency. A comprehensive literature search was executed across multiple electronic databases including Web of Science, Scopus, ERIC (Education Resources Information Center), PsycINFO, and Google Scholar to capture relevant peer-reviewed literature published between January 2020 and December 2025. This five-year window was strategically selected to focus on the contemporary landscape of generative AI tools, particularly following the widespread emergence of large language models such as ChatGPT in late 2022, while also capturing earlier automated writing evaluation systems and AI-assisted writing tools that preceded the current generative AI revolution.

The search strategy employed a combination of controlled vocabulary terms and natural language keywords organized into three thematic clusters: (1) AI technology terms including artificial intelligence, generative AI, ChatGPT, large language models, AI-assisted writing, automated writing evaluation, natural language processing, machine learning, and AI literacy; (2) educational context terms including higher education, academic writing, student writing, university, college, ESL, L2 writing, and postgraduate; and (3) assessment and pedagogy terms including assessment design, academic integrity, plagiarism, contract cheating, writing feedback, self-regulated learning, student perceptions, institutional policies, and pedagogical integration. Boolean operators (AND, OR) were utilized to combine search terms within and across clusters, with truncation symbols (*) applied to capture morphological variations (e.g., assess* to capture assessment, assessing, assessments). Database-specific subject headings and index terms were incorporated where applicable to maximize retrieval precision. To supplement the electronic database searches, forward and backward citation tracking was performed on key articles identified during initial screening, and reference lists of included systematic reviews were manually screened to identify additional relevant studies that may have been missed by the automated search. The complete search strings applied across all databases are provided in Appendix A to ensure full transparency and replicability of the search strategy.

### 2.2 Inclusion and exclusion criteria

Studies were eligible for inclusion if they met the following criteria: (1) empirical research examining the integration, use, perceptions, or impact of AI tools in academic writing contexts within higher education settings; (2) focus on writing assessment practices, evaluation strategies, pedagogical approaches, academic integrity issues, or institutional policies in the AI era; (3) inclusion of student, faculty, or institutional stakeholder perspectives on AI-assisted writing or generative AI technologies; (4) peer-reviewed journal articles, conference proceedings, or systematic reviews published in English; and (5) publication date between January 2020 and December 2025. Both quantitative and qualitative methodologies were eligible, as were mixed-methods designs and conceptual frameworks grounded in empirical evidence, to ensure comprehensive capture of diverse evidence types addressing the complex sociotechnical phenomenon of AI integration in writing assessment.

Studies were excluded if they: (1) focused exclusively on K-12 educational contexts without higher education components; (2) examined AI applications unrelated to writing or textual composition (e.g., mathematics tutoring systems, image generation tools without writing components); (3) were purely theoretical or opinion pieces without empirical data or evidence-based frameworks; (4) were published as dissertations, theses, preprints, or non-peer-reviewed sources; (5) did not address assessment, pedagogy, student learning outcomes, academic integrity, or stakeholder perspectives on AI in writing; or (6) were published in languages other than English due to resource constraints for translation and interpretation. Studies focusing solely on automated essay scoring systems from pre-2020 without contemporary generative AI context were also excluded to maintain focus on current challenges posed by large language models and generative AI technologies that emerged prominently from 2022 onwards. Table 1 presents the inclusion and exclusion criteria applied during the screening process in a structured format to facilitate transparency and replication.

**Table 1:** Inclusion and exclusion criteria for study selection.

Inclusion criteria	Exclusion criteria
Empirical research on AI integration, use, perceptions, or impact in academic writing within higher education	Studies focused exclusively on K-12 contexts without higher education components
Focus on writing assessment practices, evaluation strategies, pedagogical approaches, academic integrity, or institutional policies	AI applications unrelated to writing or textual composition (e.g., mathematics tutoring, image generation without writing components)
Inclusion of student, faculty, or institutional stakeholder perspectives on AI-assisted writing or generative AI	Purely theoretical or opinion pieces without empirical data or evidence-based frameworks
Peer-reviewed journal articles, conference proceedings, or systematic reviews published in English	Dissertations, theses, preprints, or non-peer-reviewed sources
Publication date between January 2020 and December 2025	Studies not addressing assessment, pedagogy, student learning outcomes, academic integrity, or stakeholder perspectives on AI in writing
Both quantitative and qualitative methodologies eligible, including mixed-methods designs	Publications in languages other than English; studies on pre-2020 automated essay scoring without generative AI context

### 2.3 Study selection process

The study selection process was conducted in three sequential phases following PRISMA 2020 protocols. In the initial identification phase, database searches yielded a total of 1,756 records from electronic databases (Registers: *n* = 0), with an additional 18 records identified through citation searching and manual screening of reference lists. Following deduplication (*n* = 385 duplicate records removed), 1,389 unique records remained for title and abstract screening. No records were marked as ineligible by automation tools, and no records were removed for other reasons prior to screening. During the screening phase, two independent reviewers applied the predetermined eligibility criteria, resulting in the exclusion of 1,251 records, leaving 138 full-text articles for detailed assessment. All 138 reports were successfully retrieved (Reports not retrieved: *n* = 0).

**Figure 1:**
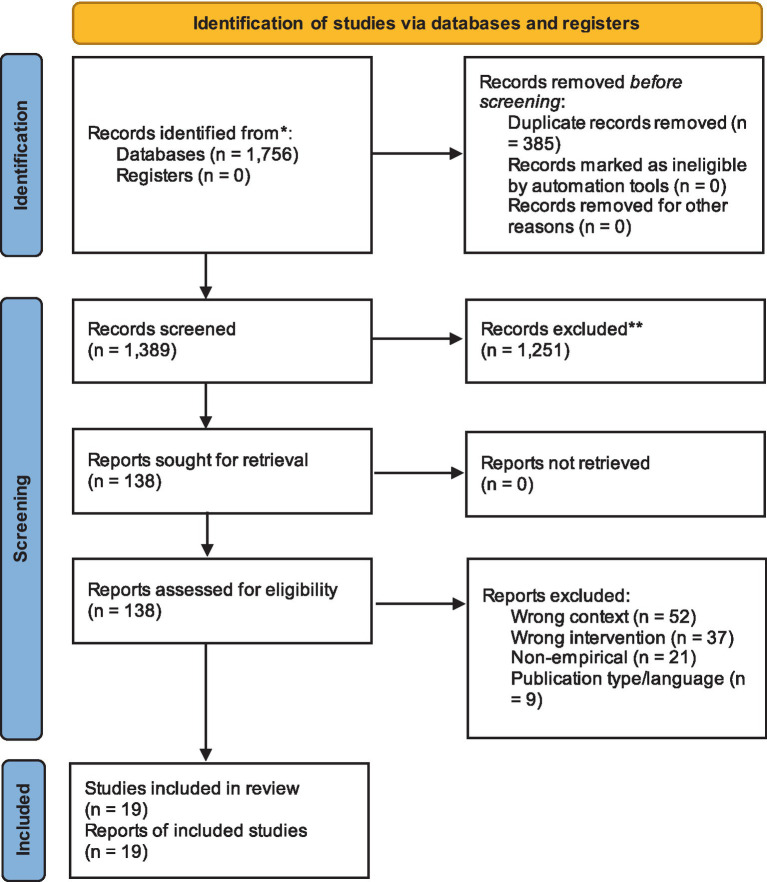
PRISMA 2020 flow diagram. Systematic review flow diagram adapted from (Page et al., 2021).

In the eligibility phase, the same two independent reviewers conducted full-text screening of all 138 retrieved articles. Reasons for exclusion were documented systematically and included: wrong educational context or level (*n* = 52), wrong intervention or focus area (*n* = 37), non-empirical studies or insufficient empirical evidence (*n* = 21), wrong publication type not meeting peer-review criteria (*n* = 6), and language restrictions (*n* = 3), totalling 119 excluded reports. Inter-rater reliability was calculated using Cohen’s kappa coefficient (*κ* = 0.87), indicating strong agreement between reviewers across the five thematic dimensions guiding data extraction: AI tool functionality, stakeholder perceptions, student motivations, institutional implementation gaps, and assessment redesign strategies. Disagreements were resolved through structured consensus discussion with reference to original study texts, and a third reviewer was available for arbitration where consensus could not be reached. Through this rigorous three-phase selection process, 19 primary studies were ultimately included in the qualitative synthesis, corresponding to 19 reports of included studies. Figure 1 presents the PRISMA 2020 flow diagram documenting the complete process.

### 2.4 Data extraction

A standardized data extraction form was developed and pilot-tested on five randomly selected included studies to ensure consistency, comprehensiveness, and reliability across reviewers. The extraction template captured multiple dimensions of study characteristics including: (1) bibliographic information (author, publication year, journal or conference venue, country of origin, institutional context); (2) study design and methodology (research design type, sample size and composition, sampling strategy, data collection methods, analytical approach and frameworks employed); (3) participant characteristics (educational level, academic discipline, demographic information where reported, linguistic background for L2 or ESL populations); (4) AI tools and technologies examined (specific tools such as ChatGPT, Grammarly, or other platforms; functional categories; usage patterns across writing stages; cognitive levels supported); (5) key findings organized by the five research question themes (tool functionality and deployment, stakeholder perceptions and attitudes, student motivations for appropriate and inappropriate use, institutional implementation gaps and challenges, assessment redesign strategies and innovations); and (6) methodological quality indicators relevant to each study design type. Data extraction was performed independently by two reviewers, with extraction tables compared systematically and discrepancies resolved through consensus discussion and reference to original study texts. For studies reporting quantitative findings, descriptive statistics, effect sizes, and statistical significance levels were extracted where available; for qualitative studies, key themes, conceptual frameworks, representative quotations, and thick descriptions were captured to facilitate rich thematic synthesis.

Given the heterogeneity of study designs, geographic contexts, student populations, and outcome measures across the 19 included studies, a narrative synthesis approach was deemed most appropriate rather than quantitative meta-analysis. Extracted data were organized thematically according to the five primary research questions guiding this review: (1) What AI tools are students and faculty using for academic writing, and what are their primary functions across different writing stages? (2) How do different stakeholder groups (students, faculty, administrators) perceive AI integration in writing assessment? (3) What factors motivate students to use AI appropriately versus inappropriately in academic writing contexts? (4) What implementation gaps exist in current institutional frameworks, policies, and support structures? and (5) What assessment redesign strategies and pedagogical innovations show promise for maintaining validity and promoting authentic skill development in the AI era? This thematic organization enabled systematic comparison of findings across diverse educational contexts, institutional settings, and student populations while preserving the nuanced insights emerging from different methodological approaches, theoretical frameworks, and geographic settings represented in the evidence base.

### 2.5 Quality assessment

A rigorous methodological quality assessment was undertaken using design appropriate critical appraisal tools to evaluate the reliability, credibility, and potential biases of the 19 included studies. Quantitative studies were evaluated with an adapted Newcastle–Ottawa Scale, while qualitative studies were appraised using the CASP checklist, and mixed methods studies with MMAT criteria. Two reviewers independently conducted all assessments, resolving disagreements through consensus. Quality ratings were applied descriptively to contextualize evidence rather than exclude studies, allowing recognition of common limitations such as reliance on convenience sampling, dependence on self reported data, limited geographic and disciplinary diversity, small qualitative sample sizes, and absence of longitudinal designs. These limitations were critically considered when interpreting the robustness and generalizability of findings, alongside indications of potential publication bias favoring positive outcomes of AI use.

### 2.6 Synthesis approach

This review used a convergent, integrated synthesis combining thematic analysis of qualitative evidence, narrative summary of quantitative results, and conceptual framework mapping. Across 19 studies, data were first deductively organized around the five guiding research questions, which structured the initial coding framework; inductive coding was then applied within each thematic category to surface emergent patterns, nuances, and sub-themes not anticipated in the original framework. This sequencing reflects the study’s planned design, in which the research questions, inclusion criteria, and extraction template necessarily shaped the analytic entry point before open-ended pattern recognition could occur. Data were then iteratively refined through constant comparison, attending to convergence and divergence by geography, institutional context, stakeholder group, disciplinary setting, and student characteristics (especially native vs. non-native English speakers). Quantitative indicators were summarized descriptively and tabulated; qualitative accounts were thematically integrated with illustrative quotations. Transparent, study-linked reporting and comprehensive evidence tables supported a configurative synthesis that advanced higher-order insights into tensions between AI support and authentic writing skill development, while foregrounding contextual moderators of applicability and transferability.

## 3 Results

This systematic review synthesized evidence from 19 primary studies to examine the integration of AI tools in academic writing assessment across diverse educational contexts. The findings are organized around five primary research questions that guided this investigation: the identification and functionality of AI tools in academic writing, stakeholder perceptions, student motivations for AI use, institutional implementation gaps, and assessment redesign strategies. Together, these findings illuminate both the opportunities and challenges inherent in balancing AI support with authentic skill development.

### 3.1 AI tools and their functions in academic writing

ChatGPT emerged as the dominant AI platform, utilized by 88.8% of students in certain samples, followed by Grammarly at 67.4% (Slimi et al., 2025). The tools identified fall into four functional categories: generative AI models (ChatGPT, Gemini, DeepSeek, Copilot) for higher-order tasks like brainstorming and drafting; automated writing evaluation systems (Grammarly, ProWritingAid, Hemingway Editor) for grammar and mechanical corrections; translation tools (DeepL, Google Translate) for cross-lingual support; and multimodal platforms (Canva, Quizlet) for visual design and vocabulary building (Amani and Bisriyah, 2025; Evmenova et al., 2024; Lancaster et al., 2025).

Students deploy these tools strategically across writing stages. LLMs dominate the planning phase for idea generation and outlining, continue supporting the drafting phase for content expansion, while AWE tools are reserved primarily for revision and editing (Wang, 2024; Zhao and Dang, 2026). Notably, LLMs support higher-order cognitive functions aligned with analysis, evaluation, and creation in Bloom’s Taxonomy, whereas AWE tools focus on lower-order skills related to remembering and understanding linguistic mechanics (Evangelista, 2025; Khlaif et al., 2025). Table 2 summarizes these tools and their primary functions.

**Table 2:** AI tools and their primary functions in academic writing.

AI tool category	Specific tools mentioned	Primary writing functions	Cognitive level supported	Frequency of use reported
Generative AI (LLMs)	ChatGPT (GPT-3.5/4.0), Gemini, DeepSeek, Copilot, Jasper	Brainstorming, idea generation, drafting, and feedback provision (Evmenova et al., 2024; Jeilani and Abubakar, 2025; Wang, 2024)	Higher-order: Analysis, creation, and evaluation (Evangelista, 2025; Wang, 2024; Zhao and Dang, 2026)	High: ChatGPT cited by 88.8% of students in some samples (Slimi et al., 2025)
Writing Assistants / AWE	Grammarly, ProWritingAid, Hemingway Editor, WordTune, Paperpal	Grammar, spelling, punctuation correction, and word choice refinement (Amani and Bisriyah, 2025)	Lower-order: Remembering and Understanding (linguistic mechanics) (Amani and Bisriyah, 2025; Khlaif et al., 2025)	High: Grammarly cited by 67.4% of students in some samples (Slimi et al., 2025)
Translation Tools	DeepL, Google Translate	Language translation and cross-lingual drafting assistance (Amani and Bisriyah, 2025; Lancaster et al., 2025; Slimi et al., 2025)	Lower-order: Linguistic translation and vocabulary retrieval (Amani and Bisriyah, 2025; Wang, 2024)	Significant among L2 and international students (Amani and Bisriyah, 2025; Wang, 2024)
Multimodal / Interactive Tools	Canva, Quizlet, Socratic, Duolingo	Visual design, vocabulary building, and research question-answering (Lancaster et al., 2025; Slimi et al., 2025)	Varied: Creating (Multimodal Digital Composing) (Lancaster et al., 2025; Slimi et al., 2025)	Canva (57.3%) and Quizlet (30.3%) reported in specific academic contexts (Slimi et al., 2025)

### 3.2 Stakeholder perceptions of AI integration

Three primary stakeholder groups—students, faculty, and administrators—exhibit markedly different perspectives on AI integration. Students demonstrate generally positive orientations, valuing AI for productivity enhancement and immediate feedback (Jeilani and Abubakar, 2025; Kumar et al., 2025). However, significant variation exists within student populations: EFL and L2 students view AI as voice-augmenting and barrier-removing, whereas native speakers report concerns about authenticity when AI suggestions diverge from personal style (Wang, 2024). Undergraduates prioritize efficiency and accessibility, while postgraduates express ethical ambiguity and concerns about voice erosion despite recognizing benefits for lexical diversity (Hanh and Duyen, 2025; Shi et al., 2025).

Faculty maintain an “integrity-centric” perspective where concerns about “AI-giarism” and critical thinking erosion overshadow perceived productivity benefits (Karkoulian et al., 2025; Lund et al., 2025). These concerns stem partly from limited familiarity with AI paraphrasing capabilities and perceived student inability to synthesize information independently (Peterson, 2025). Institutional administrators have converged on an “open but cautious” approach, rejecting total bans while emphasizing faculty-led discretion (Wang et al., 2024). A critical gap exists in resource distribution: 67% of guidance targets faculty education while only 17.8% provides direct student frameworks (Wang et al., 2024). Despite divergent perspectives, cross-stakeholder consensus supports AI functioning as a “near-peer” rather than replacing human effort, favoring process-oriented assessment (Khlaif et al., 2025). Table 3 synthesizes these stakeholder perspectives.

**Table 3:** Stakeholder perceptions of AI in writing assessment.

Stakeholder group	Primary benefits identified	Primary concerns expressed	Preferred AI integration approach	Support needs
Undergraduate Students	Increased productivity, time efficiency, and simplified learning processes (Kumar et al., 2025; Slimi et al., 2025)	Detection of AI use, reliability of AI output, and technical accessibility (Amani and Bisriyah, 2025; Wang, 2024).	AI as a “study buddy” or tutor for brainstorming and drafting assistance (Jeilani and Abubakar, 2025; Zhao and Dang, 2026).	Hands-on training, clear usage boundaries, and affordable tool access (Kumar et al., 2025; Jeilani and Abubakar, 2025; Slimi et al., 2025).
Postgraduate Students	Enhanced lexical diversity, coherence, and support for complex data manipulation (Shi et al., 2025).	Ethical ambiguity, work pressure, and potential loss of original voice (Hanh and Duyen, 2025; Wang, 2024).	Human-AI partnerships focusing on research synthesis and peer-like feedback (Zhao and Dang, 2026).	Ethics workshops, communities of practice, and mental health support (Hanh and Duyen, 2025).
ESL / L2 Students	Removal of language barriers, improved grammatical accuracy, and authorial voice augmentation (Wang, 2024).	Over-reliance leading to a decline in independent writing confidence (Amani and Bisriyah, 2025).	AI as a linguistic “scaffold” for translating and editing (Wang, 2024).	Specialized prompt engineering training and discipline-specific vocabulary support (Amani and Bisriyah, 2025; Talandis and Muller, 2025).
Faculty Members	Automated evaluation, interactive learning stimulation, and enhanced creative ideation (Evmenova et al., 2024; Karkoulian et al., 2025; Kumar et al., 2025).	Academic misconduct, uncritical acceptance of AI errors, and loss of student-teacher trust (Karkoulian et al., 2025; Kumar et al., 2025; Wang et al., 2024).	“Against, Avoid, Adopt, Explore” (AAAE) framework; process-oriented assessment (Hanh and Duyen, 2025; Khlaif et al., 2025).	Professional development, AI literacy training, and robust detection guidelines (Evangelista, 2025; Wang et al., 2024; Zhao and Dang, 2026).
Institutional Administrators	Modernized pedagogy, scalability of personalized support, and global competitiveness (Khlaif et al., 2025; Wang et al., 2024).	Data privacy, policy enforcement lag, and the “digital divide” in equity of access (Khlaif et al., 2025; Wang et al., 2024).	“Open but cautious” policies; instructor-led classroom governance (Wang et al., 2024).	Sustainable policy frameworks, ethical AI governance, and infrastructure investment (Evangelista, 2025; Jeilani and Abubakar, 2025).

### 3.3 Student motivations for AI engagement

Student engagement with AI follows two distinct pathways shaped by situational factors and institutional guidance. Appropriate usage is motivated by efficiency enhancement, cognitive load reduction, and access to personalized scaffolding (Kumar et al., 2025; Wang, 2024). Students use AI as a “walking tutor” providing instant feedback on linguistic accuracy and structural coherence, particularly during brainstorming and drafting (Talandis and Muller, 2025; Wang, 2024). This motivation is especially pronounced among non-native speakers who leverage AI to augment authorial voice and overcome communication barriers (Wang, 2024).

Inappropriate usage stems from socio-academic pressures including overloaded schedules, work pressure, and high-stakes grading systems (Hanh and Duyen, 2025; Peterson, 2025). Ethical ambiguity and institutional “policy voids” enable rationalization of full task delegation as students develop situational interpretations without explicit guidance (Hanh and Duyen, 2025; Kumar et al., 2025). AI’s technological affordances—speed, accessibility, and perceived low detection risk—create conditions where “AI-giarism” appears as a viable shortcut (Hanh and Duyen, 2025). The phenomenon of “metacognitive laziness” emerges when assessment systems emphasize final products over learning processes, incentivizing students to optimize for efficiency rather than skill development (Zhao and Dang, 2026). Notably, students engage in covert paraphrasing often due to genuine confusion about boundaries between “assistance” and “cheating” rather than malicious intent (Karkoulian et al., 2025). Table 4 analyzes motivational factors influencing usage patterns.

**Table 4:** Factors influencing AI usage patterns and academic integrity.

Motivational factor	Appropriate use manifestation	Inappropriate use manifestation	Student attribution	Institutional response strategy
Efficiency and time demands	Using AI for outlining, summarizing long texts, and brainstorming (Slimi et al., 2025; Wang, 2024; Wang et al., 2024).	Outsourcing entire assignments to save time for other obligations (Hanh and Duyen, 2025).	Intense schedules and deadline pressure (Hanh and Duyen, 2025; Peterson, 2025).	Setting word limits based on task complexity rather than credits (Zhao and Dang, 2026).
Academic Pressure	Seeking feedback to improve a draft before submission (Amani and Bisriyah, 2025).	Full delegation of tasks to ensure a “good grade” with minimal effort (Wang, 2024; Zhao and Dang, 2026).	Grade-driven expectations and social comparison (Hanh and Duyen, 2025).	Shifting to process-oriented assessment models (e.g., oral exams) (Evangelista, 2025; Khlaif et al., 2025; Peterson, 2025).
Skill Development (L2)	Using AI as a linguistic scaffold to verify grammar and word choice (Amani and Bisriyah, 2025; Wang, 2024).	Covertly paraphrasing AI content to bypass detection without learning (Hanh and Duyen, 2025; Karkoulian et al., 2025).	Language barriers and lack of confidence in academic diction (Amani and Bisriyah, 2025; Wang, 2024).	Direct instruction in prompt engineering and L2-specific AI literacy (Talandis and Muller, 2025).
Ethical Ambiguity	Acknowledging AI assistance in brainstorming or proofreading (Karkoulian et al., 2025; Wang, 2024).	Uncritical acceptance of “hallucinations” or biased output as facts (Hanh and Duyen, 2025; Wang, 2024; Zhao and Dang, 2026).	Confusion over boundaries between “assistance” and “cheating” (Hanh and Duyen, 2025).	Explicitly defining acceptable vs. prohibited uses in syllabi (Evangelista, 2025; Peterson, 2025; Wang et al., 2024).
Institutional Policy Gaps	Experimenting with AI within the parameters set by the instructor (Wang et al., 2024).	Using AI for prohibited tasks when guidelines are absent or silent (Hanh and Duyen, 2025).	Lack of formal instruction on academic integrity in the AI context (Hanh and Duyen, 2025; Wang et al., 2024).	Developing comprehensive AI governance and literacy modules (Evangelista, 2025; Kumar et al., 2025; Shi et al., 2025; Wang et al., 2024).

### 3.4 Institutional implementation gaps

Current AI integration frameworks reveal significant gaps characterized by resource disparities, training deficits, and persistent “policy voids” (Hanh and Duyen, 2025; Wang et al., 2024). Geographic inequities are pronounced: while AI tools have achieved global reach, academic discourse remains concentrated in Global North contexts, overlooking challenges in resource-constrained regions like Palestine and Somalia where political and economic limitations impede infrastructure access (Jeilani and Abubakar, 2025; Khlaif et al., 2025). Even in high-resource settings, a “digital divide” persists as disadvantaged students lack reliable internet or financial means to access premium features (Khlaif et al., 2025; Peterson, 2025).

The most critical gap is the shortage of student-facing resources: while 67% of institutional guidance targets faculty, only 17.8% provides direct student frameworks on ethical boundaries and appropriate use (Wang et al., 2024). This imbalance forces students to navigate complex decisions without pedagogical scaffolding, leading to situational interpretations and “functional failure” in distinguishing AI as learning tool versus learning substitute (Zhao and Dang, 2026). Faculty report feeling underprepared to evaluate AI-assisted work and design valid assessments, particularly those unfamiliar with modern paraphrasing capabilities (Karkoulian et al., 2025; Wang et al., 2024). Disciplinary variations compound challenges: technology departments exhibit heightened caution due to programming vulnerability, while humanities struggle when AI replicates traditional essay genres (Gonsalves, 2025; Wang et al., 2024). Institutional responses remain reactive and piecemeal, framing AI primarily as an integrity issue requiring policing rather than a pedagogical opportunity requiring curriculum redesign (Gonsalves, 2025; Hanh and Duyen, 2025). Table 5 presents evidence-based assessment redesign strategies.

**Table 5:** Assessment redesign strategies for AI-resilient writing tasks.

Assessment strategy	Theoretical framework	Cognitive skills targeted (bloom’s taxonomy)	Implementation characteristics	Effectiveness evidence
Integrated Oral Defense (Vivas)	Social Constructivism; AAAE Framework (Khlaif et al., 2025).	Evaluating, Analyzing, Creating (Evangelista, 2025).	Real-time reasoning; students must defend their written arguments orally (Evangelista, 2025).	High resistance to AI-generated shortcuts; ensures authentic understanding (Evangelista, 2025; Khlaif et al., 2025).
Product–Process Assessment	Process-Writing Workflow (Talandis and Muller, 2025).	Evaluating, Analyzing (Khlaif et al., 2025; Wang, 2024).	Evaluation focuses on iterative drafts, prompt engineering logs, and self-reflection (Khlaif et al., 2025; Talandis and Muller, 2025).	Mitigates “full delegation” by rewarding the cognitive journey over the final output (Hanh and Duyen, 2025; Wang, 2024).
Multimodal Composing	Digital Literacy Framework (Kumar et al., 2025; Shi et al., 2025).	Creating, Evaluating (Evangelista, 2025; Shi et al., 2025).	Tasks combine text, visuals, audio threads, or hand-drawn diagrams (Wang et al., 2024).	AI struggles to synthesize multiple competencies across diverse media formats (Evangelista, 2025).
Project-Based Learning (PBL)	Situated Learning Theory (Hanh and Duyen, 2025)	Analyzing, Creating (Evangelista, 2025; Khlaif et al., 2025)	Collaborative, long-term tasks addressing real-world, ill-structured problems (Evangelista, 2025; Khlaif et al., 2025).	Fosters higher-order critical thinking; identified as most cited innovative strategy (Evangelista, 2025).
Scaffolded Assignments	Cognitive Load Theory (CLT) (Zhao and Dang, 2026)	Applying, Understanding (Peterson, 2025; Wang et al., 2024)	Breaks large projects into smaller, unique tasks requiring personal knowledge links (Peterson, 2025; Zhao and Dang, 2026).	Prevents “content inflation” and uncritical reliance on AI for long outputs (Zhao and Dang, 2026).

### 3.5 Assessment redesign and policy innovations

Institutions are shifting from restrictive bans toward flexible policy frameworks balancing AI affordances with academic rigor (Wang et al., 2024). The “Against, Avoid, Adopt, and Explore” (AAAE) framework from Palestine provides task-based categorization ranging from prohibited use in traditional exams to full human-AI partnership in creative problem-solving (Khlaif et al., 2025). The “Six Domains of Academic Integrity Reporting” (AIR) emphasizes coaching and literacy education, distinguishing deliberate cheating from “functional failure” in synthesis skills (Peterson, 2025). Most U.S. universities adopted “open but cautious” stances with faculty-led discretion for discipline-specific relevance (Wang et al., 2024).

To maintain validity, institutions prioritize assessments targeting higher-order cognitive skills AI struggles to replicate (Evangelista, 2025). Real-time oral defenses ensure authentic understanding by requiring extemporaneous argument articulation (Evangelista, 2025; Khlaif et al., 2025). Process-oriented assessment shifts focus from final products to iterative drafts, prompt engineering logs, and reflective exercises documenting cognitive journeys (Khlaif et al., 2025; Talandis and Muller, 2025; Wang, 2024). Multimodal composing requiring integration of text, visuals, and audio resists AI synthesis capabilities (Evangelista, 2025). Project-based learning engaging real-world problems promotes critical thinking beyond AI’s current capabilities (Evangelista, 2025; Khlaif et al., 2025). Explicit instruction in prompt engineering and critical AI literacy positions AI as legitimate skill development object rather than taboo (Talandis and Muller, 2025; Wang, 2024). Table 6 illustrates geographic variations in implementation.

**Table 6:** Geographic and institutional variations in AI integration.

Geographic region/context	Institutional AI policy approach	Key challenges identified	Digital literacy level	Recommended framework applied
Global North (U.S. Top 100)	“Open but cautious”; faculty-led discretion (Wang et al., 2024).	Significant resource gap for student guidance; data privacy concerns (Wang et al., 2024)	High awareness but lacks systematic pedagogical integration (Wang et al., 2024).	Technological Pedagogical Content Knowledge (TPACK) (Wang et al., 2024).
Middle East (Palestine)	AAAE Model; focus on AI-resistant assessment design (Khlaif et al., 2025).	Digital divide, political limitations, and lack of professional development (Khlaif et al., 2025).	Emerging; focused on bridging the gap between novice and expert use (Khlaif et al., 2025).	Against, Avoid, Adopt, Explore (AAAE) (Khlaif et al., 2025).
East Africa (Somalia)	Perceived institutional support based on social cognitive needs (Jeilani and Abubakar, 2025).	Limited technological infrastructure; low student confidence in high-tech tasks (Jeilani and Abubakar, 2025).	Predictor of technology self-efficacy; needs hands-on training (Kumar et al., 2025; Jeilani and Abubakar, 2025).	Social Cognitive Theory (SCT) (Jeilani and Abubakar, 2025).
Southeast Asia (Vietnam)	Developmental/Exploratory; focus on postgraduate ethical reasoning (Hanh and Duyen, 2025).	Ethical ambiguity, work pressure, and uncritical acceptance of AI content (Hanh and Duyen, 2025).	Varies by academic level; risk of “metacognitive laziness” (Hanh and Duyen, 2025; Zhao and Dang, 2026)	Dual-Pathway (Top-Down Governance & Bottom-Up Culture) (Hanh and Duyen, 2025).
Middle East (Lebanon)	Qualitative assessment of productivity vs. integrity (Karkoulian et al., 2025).	Faculty unawareness of paraphrasing tools; prioritization of convenience over ethics (Karkoulian et al., 2025).	Nuanced; many students use AI for “disguising” generated content (Karkoulian et al., 2025).	Integrated Ethical AI Education (Karkoulian et al., 2025).

### 3.6 Publication and methodological bias assessment

Following PRISMA 2020 guidelines, publication bias assessment revealed a “positive-utility” reporting bias favoring successful implementations while null or negative findings remain scarce (Kumar et al., 2025; Shi et al., 2025). Sullivan et al. (2023) identified systemic marginalization of student voices in favor of institutional integrity narratives. The evidence base may reflect “time-lag bias” where early success stories receive publication priority while longer-term outcomes remain undocumented (Wang et al., 2024).

Methodological scrutiny revealed that large-scale surveys employed rigorous controls and theoretical grounding (Jeilani and Abubakar, 2025; Lund et al., 2025), but substantial evidence relies on convenience sampling and self-reported data introducing potential social desirability bias regarding misconduct behaviors (Amani and Bisriyah, 2025; Hanh and Duyen, 2025; Karkoulian et al., 2025). Geographic diversity across Global North and South contexts strengthens external validity (Hanh and Duyen, 2025; Jeilani and Abubakar, 2025; Khlaif et al., 2025). Secondary systematic reviews provide triangulation reducing single-study outlier influence (Evangelista, 2025). The absence of longitudinal data limits determination of long-term impacts on critical thinking skills (Zhao and Dang, 2026).

## 4 Discussion

The stratified deployment of AI tools across cognitive complexity levels points to a deeper developmental paradox: while students demonstrate growing strategic sophistication in managing cognitive load, this very efficiency may be systematically displacing the productive struggle through which genuine writing expertise is built (Sweller, 1988; Zhai et al., 2024). The dominance of generative AI platforms like ChatGPT (88.8%) and traditional writing assistants like Grammarly (67.4%) reflects students’ differentiated cognitive needs throughout the writing process (Slimi et al., 2025). This stratified deployment aligns with cognitive load theory, suggesting students strategically offload routine mechanical tasks to preserve cognitive resources for complex reasoning and argumentation. However, this functional division raises critical concerns about authentic skill development. The pattern of consistent delegation—grammar to automated systems, ideation to generative models—may create a developmental paradox where students gain surface-level facility with AI-mediated composition while failing to internalize the underlying competencies that constitute genuine writing expertise and independent authorial capacity (Zhai et al., 2024).

This divergence is not simply a matter of competing preferences but reflects fundamentally incompatible assumptions about what constitutes legitimate intellectual work—assumptions that existing policy frameworks have yet to adequately reconcile (Karkoulian et al., 2025; Wang et al., 2024). Students, particularly those facing language barriers or socioeconomic pressures, conceptualize AI as an equalizing tool that democratizes access to high-quality writing support (Wang, 2024). Faculty members, conversely, maintain what might be termed an “integrity-centric” worldview where concerns about assessment validity and intellectual authenticity overshadow potential pedagogical benefits (Karkoulian et al., 2025; Lund et al., 2025). The institutional response has been notably asymmetric, with 67% of guidance disproportionately targeting faculty professional development while only 17.8% addresses students directly, leaving them to navigate ethical complexities without adequate scaffolding (Wang et al., 2024).

The evidence challenges simplistic narratives that attribute inappropriate AI use to character flaws or academic dishonesty. Instead, a more nuanced picture emerges where contextual pressures, institutional policy voids, and technological affordances converge to create what might be termed “structural vulnerability” to AI misuse. Students operating under intense time constraints, managing multiple competing obligations, and facing high-stakes assessment systems make pragmatic decisions where immediate survival needs override abstract commitments to long-term skill development (Hanh and Duyen, 2025; Peterson, 2025). Particularly significant is the finding that many students engage in unauthorized AI use not from malicious intent but from genuine uncertainty about boundaries between legitimate support and prohibited delegation. This distinction between deliberate cheating and what Peterson (2025) has termed “functional failure” has profound implications for institutional responses, suggesting that punitive detection-focused approaches may be addressing symptoms rather than underlying causes.

Geographic analysis reveals a troubling pattern where AI integration may be amplifying rather than reducing educational inequalities across global contexts. While technological tools theoretically offer universal accessibility, their meaningful pedagogical integration depends critically on infrastructure reliability, institutional capacity for innovation, and economic resources to access premium functionalities. Students in under-resourced contexts like Palestine and Somalia face connectivity limitations and political constraints that fundamentally impede access (Jeilani and Abubakar, 2025; Khlaif et al., 2025). The concentration of research, policy development, and pedagogical expertise in Global North contexts creates an epistemological asymmetry where frameworks and best practices reflect particular cultural assumptions about authorship, collaboration, and intellectual property that may not translate seamlessly across diverse educational traditions (Chan, 2023; Gustilo et al., 2024). Even within relatively wealthy nations, historical patterns of institutional stratification are being reproduced in the AI era, with elite universities demonstrating sophisticated integration while under-resourced institutions struggle with basic access and support infrastructure (Maimela and Mbonde, 2025).

Several limitations constrain the generalizability and robustness of current findings. The review’s conclusions are further constrained by the relatively small corpus of 19 included studies, which limits the breadth of evidence and the weight that can be placed on any emerging pattern. The predominance of convenience sampling and reliance on self-reported data introduce potential biases, particularly around sensitive topics like academic misconduct where social desirability effects may distort reporting. The absence of longitudinal research designs means that assertions about long-term impacts on critical thinking, independent problem-solving, and professional competence remain largely speculative rather than empirically grounded. Geographic concentration of research effort creates significant blind spots regarding how AI integration unfolds in diverse institutional and cultural contexts. Future reviews should aim to expand the evidence base to strengthen the transferability of these findings. Perhaps most critically, the rapid evolution of AI capabilities introduces temporal instability where findings about current system limitations may quickly become obsolete.

## 5 Conclusion

This systematic review of 19 empirical studies yields five interconnected findings that collectively reframe how AI integration in academic writing assessment should be understood. First, students deploy AI tools in a stratified, cognitively strategic manner—using generative models such as ChatGPT for higher-order planning and drafting while reserving automated writing evaluation tools for mechanical revision, a pattern reflecting deliberate cognitive load management rather than indiscriminate use. Second, stakeholder perspectives diverge fundamentally: students prioritize accessibility and productivity, faculty foreground integrity and assessment validity, and administrators adopt cautious openness while disproportionately directing guidance toward faculty (67%) over students (17.8%). Third, inappropriate AI use is driven not by academic dishonesty but by structural vulnerabilities—time pressure, ethical ambiguity, and institutional policy voids—a distinction with direct implications for how institutions should respond. Fourth, geographic and socioeconomic inequities are being amplified rather than resolved by AI adoption, with Global South institutions facing infrastructure and policy deficits that widen existing educational disparities. Fifth, promising assessment innovations including oral defenses, process-oriented evaluation, multimodal composing, and explicit AI literacy curricula offer viable pathways for maintaining assessment validity while supporting authentic skill development.

The evidence reveals significant implementation gaps including resource asymmetries, geographic inequities, and policy frameworks that inadvertently incentivize the very behaviors they seek to prevent. Yet promising innovations are emerging that suggest viable pathways forward: flexible policy architectures that recognize contextual variation, process-oriented assessment that privileges learning over products, and explicit AI literacy curricula that position technological competence as legitimate educational outcome. Taken together, these findings suggest that the central challenge is not technological but pedagogical and ethical: institutions must move from reactive policing of AI use toward proactive curriculum redesign that positions critical judgment, ethical reasoning, and authentic authorship as the irreducible core of academic writing education. Success requires moving beyond binary framings of AI as either threat or panacea toward nuanced integration grounded in equity, focused on authentic learning, and committed to developing the distinctly human capacities for critical judgment, creative synthesis, and ethical reasoning that remain essential regardless of technological advancement.

## Data Availability

The original contributions presented in the study are included in the article/Supplementary material, further inquiries can be directed to the corresponding author.
