# Supplementary material for: Reimagining writing assessment for the AI era: a systematic review on balancing AI support and authentic skill growth
Source: Front Psychol. 2026 May 26;17:1809174. doi: 10.3389/fpsyg.2026.1809174 (PMC13246662; doi:10.3389/fpsyg.2026.1809174)
Supplement: Supplementary file 1 [file Table_1.docx]

# Appendix A: Search Strategy

# A1. Complete Boolean Search Strings by Database

The following table presents the complete Boolean search strings applied across all five electronic databases during the systematic literature search. Search terms were organized into three thematic clusters: (1) AI technology terms, (2) educational context terms, and (3) assessment and pedagogy terms. Boolean operators (AND, OR) were used to combine terms within and across clusters, with truncation symbols (*) applied to capture morphological variations.

| **Database** | **Search String** |
| --- | --- |
| **Web of Science** | (TS=(("artificial intelligence" OR "generative AI" OR "ChatGPT" OR "large language model*" OR "AI-assisted writing" OR "automated writing evaluation" OR "natural language processing" OR "machine learning" OR "AI literacy") AND ("higher education" OR "academic writing" OR "student writing" OR "universit*" OR "college" OR "ESL" OR "L2 writing" OR "postgraduate") AND ("assessment design" OR "academic integrity" OR "plagiarism" OR "contract cheating" OR "writing feedback" OR "self-regulated learning" OR "student perception*" OR "institutional polic*" OR "pedagogical integration"))) |
| **Scopus** | (TITLE-ABS-KEY(("artificial intelligence" OR "generative AI" OR "ChatGPT" OR "large language model*" OR "AI-assisted writing" OR "automated writing evaluation" OR "natural language processing" OR "machine learning" OR "AI literacy") AND ("higher education" OR "academic writing" OR "student writing" OR "universit*" OR "college" OR "ESL" OR "L2 writing" OR "postgraduate") AND ("assessment design" OR "academic integrity" OR "plagiarism" OR "contract cheating" OR "writing feedback" OR "self-regulated learning" OR "student perception*" OR "institutional polic*" OR "pedagogical integration"))) AND PUBYEAR > 2019 AND PUBYEAR < 2026 AND DOCTYPE(ar OR cp OR re) |
| **ERIC** | (("artificial intelligence" OR "generative AI" OR "ChatGPT" OR "large language models" OR "AI-assisted writing" OR "automated writing evaluation" OR "AI literacy") AND ("higher education" OR "academic writing" OR "college students" OR "ESL" OR "second language writing") AND ("writing assessment" OR "academic integrity" OR "plagiarism" OR "writing feedback" OR "student perceptions" OR "institutional policy")) AND (Publication Date: 2020-2025) |
| **PsycINFO** | (("artificial intelligence" OR "generative AI" OR "ChatGPT" OR "large language model*" OR "automated writing evaluation" OR "AI literacy") AND ("higher education" OR "academic writing" OR "university students" OR "ESL" OR "L2 writing") AND ("writing assessment" OR "academic integrity" OR "writing feedback" OR "self-regulated learning" OR "student motivation" OR "pedagogical integration")) AND PY(2020-2025) |
| **Google Scholar** | "artificial intelligence" OR "generative AI" OR "ChatGPT" AND "academic writing" OR "writing assessment" AND "higher education" OR "university" AND "academic integrity" OR "writing feedback" OR "student perceptions" 2020-2025 |
